# Response to collective threat: Russian invasion unifies Ukrainians across ethnic, linguistic, religious and geographic lines

**DOI:** 10.1098/rsos.241005

**Published:** 2025-01-22

**Authors:** Joshua Borycz, Benjamin D. Horne, Catherine Luther, Garriy Shteynberg, Suzie Allard, Brandon Prins, R. Alexander Bentley

**Affiliations:** ^1^ Stevenson Science and Engineering Library, Vanderbilt University, Nashville, TN 37203, USA; ^2^ School of Information Sciences, University of Tennessee, Knoxville, TN 37996, USA; ^3^ School of Journalism and Media, University of Tennessee, Knoxville, TN 37996, USA; ^4^ Department of Psychology, University of Tennessee, Knoxville, TN 37996, USA; ^5^ Department of Political Science, University of Tennessee, Knoxville, TN 37996, United States; ^6^ Department of Anthropology, University of Tennessee, Knoxville, TN 37996, USA

**Keywords:** social norms, social identity, intergroup conflict, political polarization, collective threat

## Abstract

Under the collective threat of war, the 2022 Russian invasion would be expected to unify Ukrainians across distinct ethnic, linguistic, geographic and generational identities. Here, we show this using survey data collected in Belarus and Ukraine before and after the full-fledged invasion of Ukraine by Russia. Using our data collection waves from spring and summer of 2022, we observed attitudinal changes rarely documented before and after such an event. Our data include both the invaded country, Ukraine, as the ‘treatment’ and a non-invaded country, Belarus, as the ‘control’. We find that, in Ukraine but not in Belarus, geopolitical views were sharply unified by the experience of the invasion, outweighing the heterogeneous group identities before the event. Our observations serve as evidence that identity fusion under collective threat can override long-standing social divisions.

## 1. Introduction

Human groups under collective threat develop tighter cultural norms [1], wherein stricter social rules, monitoring and punishment increase group coordination and uniformity [2–4]. As a result of shared personally transformative experiences, this coordination, or identity fusion [5], cuts across the social identities that normally underlie human beliefs, emotions and behaviours [6–9]. Identifying with a given ethnic, religious and/or linguistic group aligns an individual’s psychology and behaviour to its prevailing social norms [1,10,11]. Although these norms are known to evolve slowly, over generations of demographic, socio-political and cultural change [12–16], the influence of social identities can also be more dynamic [17], with turnover in social identities that underlie peoples’ beliefs.

Under collective threat, social identities of ethnicity, religion and language may lose their influence over beliefs and attitudes. Against perceived out-group threat, in-group cohesion is a deeply evolved response not only among humans [18] but also among chimpanzees [19–21], certain social animals [22], birds [23] and even fish [24]. Among humans, warfare was prominent as an out-group threat by the early Holocene [25]. The collective experience of war, through shared awareness [26,27] and shared dysphoric experience [28], can sap the influence of previously meaningful social identities via the tightening of social norms [3].

A potential example of this follows the full-fledged invasion of Ukraine by Russia in February 2022. Surveys in 2022 indicate that Ukrainians remained motivated to resist the invading forces and that this was a unifying factor for Ukrainians [29]. As Russian troops were preparing to invade, journalists in Ukraine described ‘A people long divided by profound disputes over what language to speak, what church to follow and what historical heroes to revere has begun to stitch together a sense of common purpose in the face of a menacing foe’ [30].

**Figure 1.**
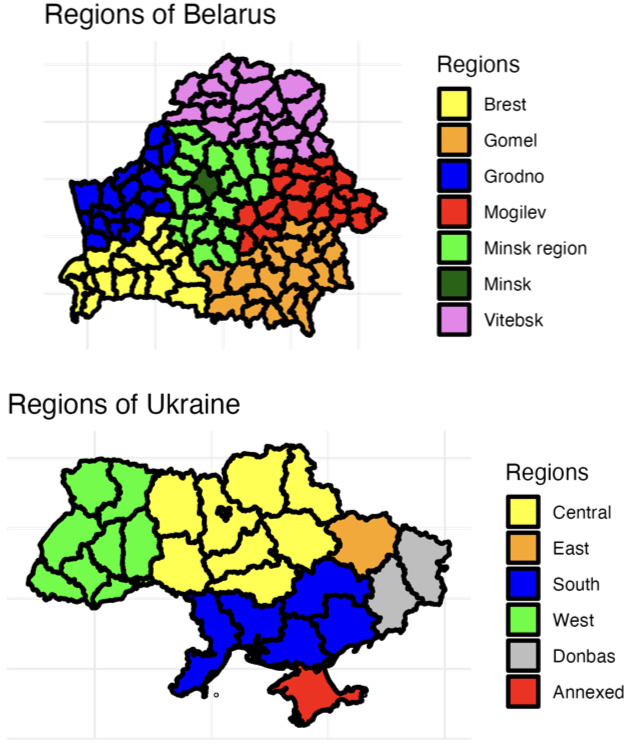
Regions of Belarus and Ukraine used in the surveys. The Donbas region and the annexed region of Crimea were not included in the surveys.

This unification of purpose was not a foregone conclusion, however, because the support of Russia still existed amongst the Russian-speaking populations in Ukraine even after Russia’s annexation of Ukraine’s Crimean Peninsula in 2014 [31], and the Kremlin’s backing of pro-Russian separatists in the Donbas region of eastern Ukraine (figure 1). Annual surveys in Latvia from 2022 to 2024 reveal that, whereas over 80% of surveyed Latvian speakers blamed Russia for the war in Ukraine, less than 40% of the Russian speakers blame Russia [32]. This polarization among Latvia’s Russian speakers, which include ethnic Ukrainians and other groups, exists despite the risks the war poses to their national security [32].

Here, we present a serendipitous ‘natural experiment’ [33,34] involving the Russian–Ukrainian war and our ongoing survey project in the Former Soviet Republics (FSRs) of Ukraine and Belarus in 2021 and 2022. Our study observes the effect on opinions in two neighbouring FSRs across an event, with Ukraine serving as the ‘treatment’ condition and Belarus the quasi-control condition. While not identical, Ukraine and Belarus are linguistically and culturally similar FSRs, and each has been subjected to decades of Russian propaganda.

Our access to opinions in these countries follows a larger project, which has examined the effectiveness of Russian propaganda narratives in shaping public opinion in select FSRs [35]. Because we began these surveys before the invasion of Ukraine, we have unique observational datasets for how opinions changed from spring to summer of 2022, before and after the event, respectively.

## 2. Methods

Our data consist of two representative surveys administered to random samples of Russian-speaking people from Belarus and Ukraine in two waves, spring of 2022 and summer of 2022 (table 1). During the first wave, the surveys were administered in Belarus (n=1001) from 21–22 February 2022, and in Ukraine (n=2053) from 18–23 February 2022, immediately prior to Russia’s invasion of Ukraine on 24 February 2022. During the second wave, the surveys were administered in Belarus (n=1004) from 30–31 July 2022, and in Ukraine (n=1007), from 6–7 August 2022. In the first wave, the surveys were administered through a CATI method in Belarus due to the political situation in the country at that time and for Ukraine, the surveys were administered in person. During the second wave, with Russia’s invasion of Ukraine, surveys were administered solely by the CATI method. Respondents were permanent residents and were at least 18 years of age (table 2). The samples were representative of the general populations of each country by age, gender, region and size of settlement. In Ukraine, the Donbas region and Crimea were not included in the sample, as geopolitical events hindered our access to these regions for our surveys.

**Table 1.** Number of participants per region per wave in Ukraine and Belarus used in our analysis.

Ukraine			Belarus		
region	pre-invasion	post-invasion	region	pre-invasion	post-invasion
Central	684	412	Minsk	242	234
West	598	190	Minsk (region)	136	168
South	508	252	Vitebsk	143	115
East	263	153	Brest	149	130
­	­	­	Gomel	127	142
­	­	­	Mogilev	104	115
­	­	­	Grodno	100	100
Total	2053	1007	Total	1001	1004

**Table 2.** Percentage of adult respondents by age range and self-described knowledge of the Russian language per wave in Ukraine and Belarus.

Ukraine			Belarus	
	pre-invasion	post-invasion	pre-invasion	post-invasion
Age				
18–24	8.6%	7.9%	7.6%	6.6%
25–34	19.8%	18.0%	18.3%	14.8%
35–44	18.9%	18.7%	16.5%	17.8%
45–54	16.6%	19.0%	15.9%	21.0%
55–64	19.5%	18.6%	20.7%	18.4%
65 and over	16.6%	17.9%	21.1%	21.3%
Russian				
none	1.2%	2.1%	0.1%	0.2%
read	7.3%	9.2%	2.3%	2.1%
communicate	7.8%	5.2%	2.1%	1.9%
understand	2.1%	2.1%	0.4%	0.4%
fluent	81.7%	81.3%	95.0%	95.4%

To demonstrate the consistency in our samples across these two waves, table 3 shows the number and percentage of respondents by self-identified religion for each country pre- and post-invasion. In Belarus, the percentages are consistent before and after the invasion. In Ukraine, there is a moderate increase in the percentage identifying as Orthodox and a decrease in Muslim and Catholic by several per cent each (table 3). These shifts in Ukraine, all less than 10%, are not unexpected after an invasion, and could be due to movement of people and/or changes in self-identification of religious affiliation.

**Table 3.** Number and percentage of participants self-identifying by religion (or declining to answer) per wave in Ukraine and Belarus.

Ukraine			Belarus	
Religion	pre-invasion	post-invasion	pre-invasion	post-invasion
Orthodox	1235 (60%)	690 (69%)	787 (79%)	788 (78%)
Catholic	215 (10%)	49 (5%)	72 (7%)	80 (8%)
Muslim	154 (8%)	53 (5%)	4 (0.4%)	3 (0.3%)
Secular	10 (0.5%)	10 (1%)	93 (9%)	113 (11%)
No response	439 (21%)	205 (20%)	45 (4%)	20 (2%)
Total	2053	1007	1001	1004

The dependent variable in this study is who the Russian-speaking populations of Belarus and Ukraine blame for the tension/war between Russia and Ukraine before and after the war in Ukraine began. The survey questions, limited in number, aimed to assess views of the Russian government and its competence. We asked respondents who they thought were to blame for tensions between Russia and Ukraine: ‘Who do you think is responsible for the worsening tensions between Russia and Ukraine?’ (called *blame* henceforth). We also asked if Russian policies benefit or hurt their country: ‘In general, how do Russian policies affect your country?’ (called *policy* henceforth). Though limited by the logistics of access, these questions addressed attitudes towards the Russian government from various angles, and small focus group interviews were also conducted to verify the relationships identified in the larger surveys.

## 3. Results

We view the results in two countries, Belarus and Ukraine, at two times, in the spring of 2022 and in the summer of 2022, before and after the invasion. The results are aggregated across different regions (figure 1) and demographic and socio-cultural covariates, in terms of what fraction of respondents replied that Russia is to blame for the war. As a robustness check, we also report the fraction who believe Russian policy is having a positive versus a negative effect on their country, respectively.

We perform nine two-way analysis of variance tests (ANOVA, using *stats* in R v. 4.3.1) across our nine demographic variables of interest with *blame* as the dependent variable and the season as the interaction term, where season is when the survey took place. The two-way ANOVA results in table 4 summarize the effect of demographics and the timing of the survey (before versus after the invasion) on whether Russia was blamed for the tension/war between Ukraine and Russia. In Ukraine, the most notable effect is the heterogeneity in geopolitical views before the invasion of Ukraine, followed by a dramatic shift towards blaming Russia after the invasion, particularly across subgroups of religion, language, region and nationality (table 4). None of these shifts are seen in Belarus (table 4).

**Table 4.** ANOVA F-values for the impact of season (pre- and post-invasion of Ukraine) and demographics (X) on blame for tensions between Ukraine and Russia.

	Belarus: blame (tensions)			Ukraine: blame (tensions)		
	X	season	X:season	X	season	X:season
age	54.5***	17.0***	0.2	1.4	306.4***	0.5
gender	0.9	13.1***	0.0	1.6	304.9***	0.3
education	16.5***	13.8***	1.7	5.1**	289.9***	0.5
region	13.2***	14.6***	1.4	69.5***	367.1***	23.0***
nationality	1.2	13.2***	0.3	22.1***	306.6***	4.3**
employment	24.8***	12.6***	0.7	3.4**	298.5***	0.3
wealth	3.8**	12.2***	0.9	12.3***	273.5***	1.5
religion	30.5***	12.1***	0.2	87.7***	218.4***	3.4*
language	1.9	13.3***	0.1	6.1**	302.8***	4.0**

Significance: ***p<0.001, **p<0.01, *p<0.05.

To corroborate these patterns, we performed the same analysis on attitudes towards Russian policy in Ukraine versus Belarus (pre- and post-invasion). The ANOVA in table 5 shows that the primary effects of region, nationality and religion on *policy* were significantly moderated by the season in which the survey was taken, matching the results found for *blame* in table 4. In the case of Belarus, the demographics alone have some relationship with the outcome, but those demographics’ interaction with the intervention of the war (X:Season) does not. After the invasion, demographic differences in blame persisted more in Belarus than in Ukraine.

**Table 5.** ANOVA F-values for impact of season (pre- and post-invasion of Ukraine) and demographics (X) on blame for perceived effect of Russian policy.

	Belarus: blame (policy)			Ukraine: blame (policy)		
	season	X	X:season	season	X	X:season
age	16.9***	22.7***	0.9	2.1	71.7***	0.9
gender	2.6	19.1***	0.1	0.1	71.6***	1.8
education	16.5***	19.0***	0.8	2.0	69.6***	0.2
region	12.4***	20.8***	2.4*	38.0***	91.8***	28.3***
nationality	1.0	19.3***	0.8	13.7***	71.1***	2.6*
employment	12.6***	17.1***	1.4	2.2*	71.2***	1.5
wealth	4.1***	17.9***	0.9	3.2**	67.1***	1.5
religion	23.7***	18.8***	1.4	69.4***	34.0***	10.0***
language	2.7*	18.7***	0.2	4.0**	68.9***	1.7

Significance: ***p<0.001, **p<0.01, *p<0.05

**Figure 2.**
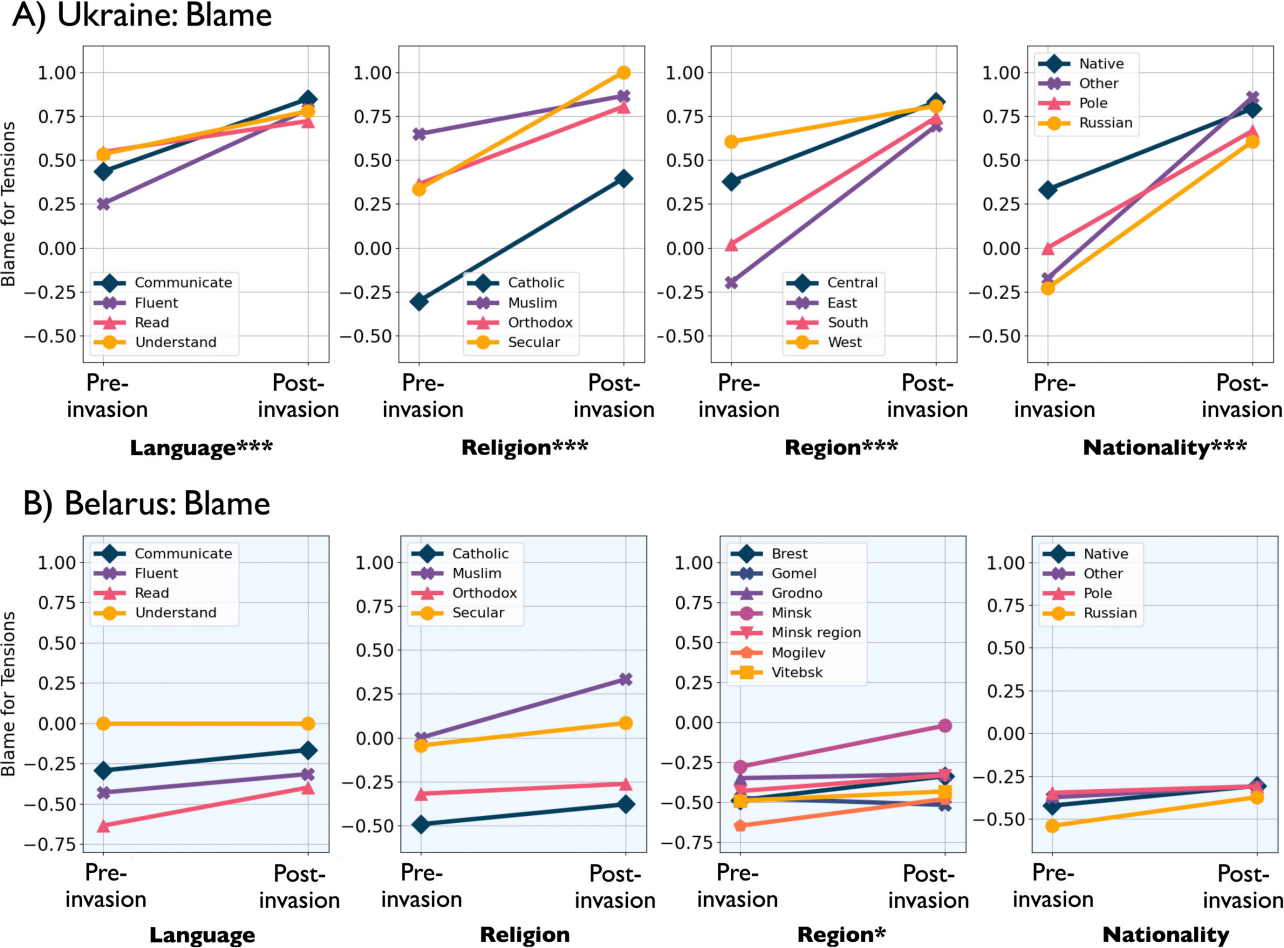
Interaction plots showing mean change in blame pre- and post-invasion in (*a*) Ukraine and (*b*) Belarus, where closer to 1 is blame Russia and closer to −1 is blame USA/NATO/Ukraine (West). If the interaction between the demographic variable and the impact of war is significant, this is noted with the following codes: *** p<0.001, ** p<0.01, * p<0.05.

This contrast in effect between Ukraine and Belarus is also seen in variance compression across multiple covariates. Table 6 shows that, in Ukraine, the standard deviation in blame attribution decreased after the invasion within each demographic subgroup among Russian speakers, Orthodox religion members, residents of all four regions and among both native and Russian-born Ukrainians. We see none of these shifts in Belarus (table 7). When examining change in the variance of blame attribution across demographic subgroups (rather than within each subgroup), we see a similar pattern. In Ukraine, the variance of blame attribution across varying levels of Russian language proficiency and across all regions was significant before the invasion but insignificant after (Fligner-Killeen tests, table 8). The exception to this rule is across religious subgroups, which reflects the still high variance of blame attribution among Catholics in our sample. Importantly, as shown in figure 2*b* and table 6, the blame attribution of Catholics in Ukraine *does* shift towards blaming Russia after the invasion (see also figure 6); the variance of those responses simply remains higher than the responses of other subgroups. Overall, this suggests a unification of views across demographic categories.

**Table 6.** Standard deviation of blame within groups before and after invasion in Ukraine.

Demographic	Ukraine: standard deviation, pre- and post-invasion							
Russian language	Fluent***		Communicate***		Read*		Understand	
	0.835	0.464	0.747	0.394	0.661	0.529	0.699	0.426
Religion	Orthodox***		Secular*		Catholic		Muslim*	
	0.791	0.435	0.800	0.000	0.819	0.664	0.595	0.407
Region	East***		West***		Central***		South***	
	0.807	0.491	0.656	0.422	0.779	0.444	0.867	0.529
Nationality	Native***		Russian***		Polish		other**	
	0.804	0.464	0.822	0.560	0.942	0.471	0.843	0.547

Significance from Fligner–Killeen test pre- and post-invasion within each subgroup: ***p<0.001, **p<0.01, *p<0.05

**Table 7.** Standard deviation of blame within groups before and after invasion in Belarus.

demographic	Belarus: standard deviation, pre- and post-invasion							
Russian lang.	fluent		communicate		read		understand	
	0.661	0.674	0.699	0.586	0.488	0.583	0.000	0.707
Religion	Catholic		Secular		Orthodox		Muslim	
	0.684	0.685	0.716	0.682	0.630	0.650	0.707	0.471
Region	Minsk		Minsk region		Vitebsk		Grodno	
	0.727	0.749	0.660	0.620	0.613	0.646	0.656	0.633
	Brest		Mogilev		Gomel			
	0.630	0.641	0.543	0.608	0.613	0.566		
Nation- ality	native		Russian		Polish		other	
	0.658	0.667	0.618	0.706	0.624	0.650	0.732	0.650

Significance from Fligner–Killeen test pre- and post-invasion within each subgroup: *** p<0.001, **p<0.01, *p<0.05

**Table 8.** Fligner–Killeen tests for equality of variance of blame across subgroups within each demographic in Belarus and in Ukraine.

	Belarus		Ukraine	
demographic	pre-	post-	pre-	post-
Russian language	4.72	2.76	25.90***	2.51
Religion	0.286	4.89	23.25***	41.38***
Region	9.09	6.18	59.61***	8.51
Nationality	3.94	0.709	0.12	5.01

Significance: ***p<0.001, **p<0.01, *p<0.05.

**Figure 3.**
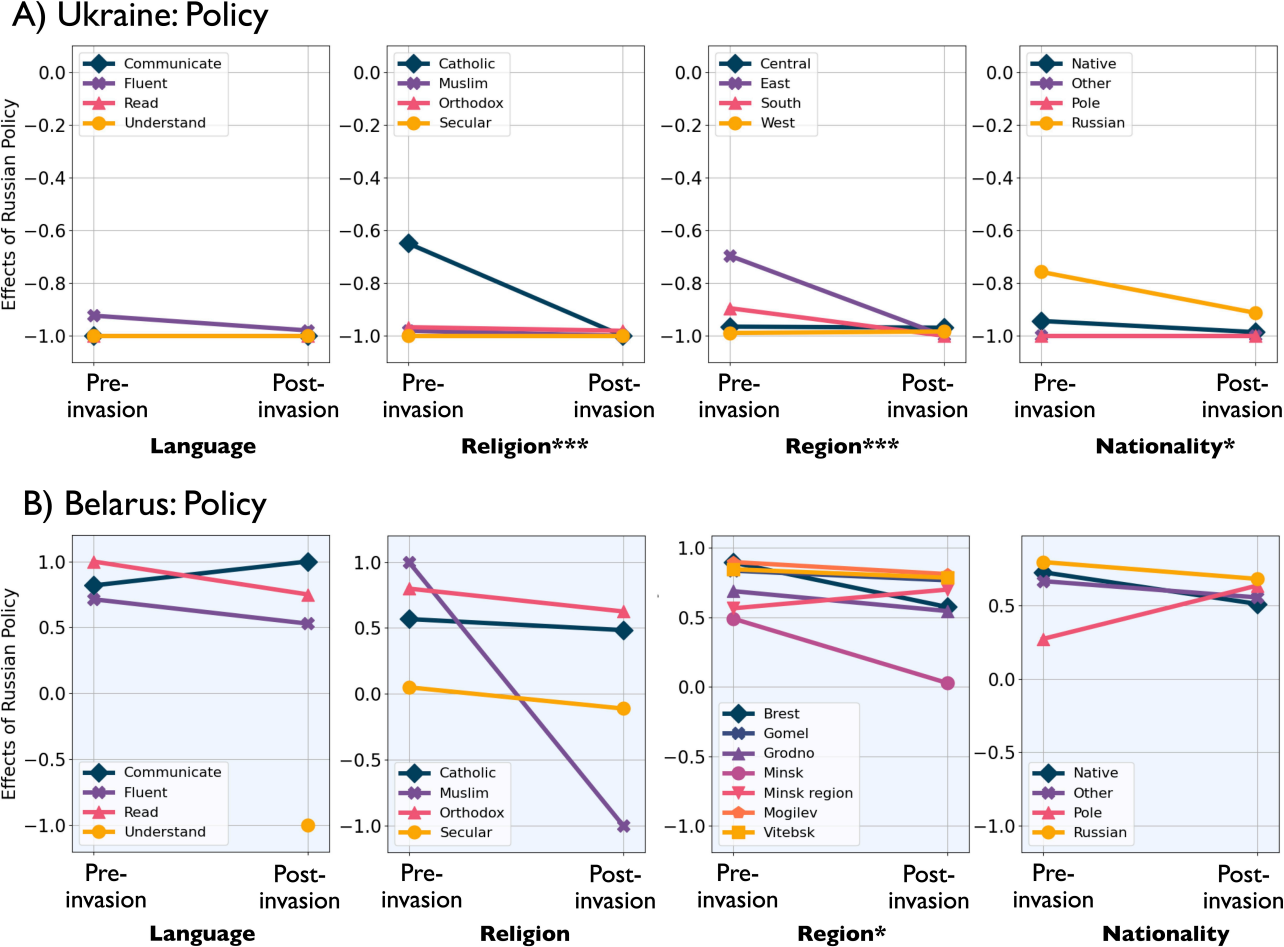
Interaction plots showing mean change in Russian policy affects pre- and post-invasion in (a) Ukraine and (b) Belarus, where closer to 1 is the opinion that Russian policy benefits the country and closer to −1 is the opinion that Russian policy hurts the country. If the interaction between the demographic variable and the impact of war are significant, this is noted with the following codes: *** p<0.001, ** p<0.01, * p<0.05.

**Figure 4.**
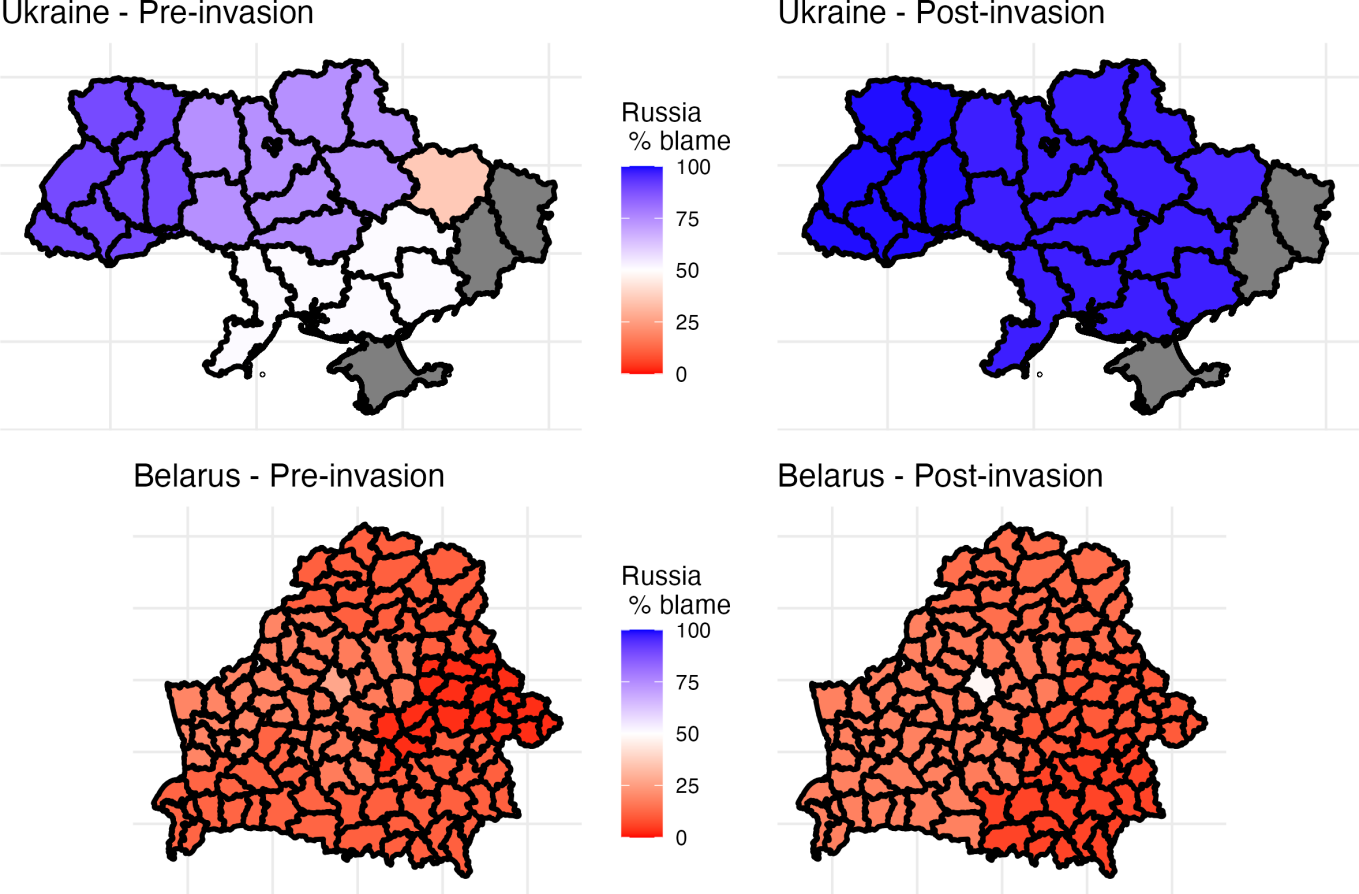
Change in blame in the regions of Ukraine and Belarus before and after the invasion of Ukraine (grey: no data).

These changes are more apparent graphically in the interaction plots of figures 2 and 3. Before the war began, there were differences between regions of Ukraine in their level of blame towards Russia or NATO/USA/Ukraine. Eastern regions of Ukraine were more likely to blame the USA/NATO/Ukraine, while western regions blamed Russia more (figure 4). After the war began, however, all regions overwhelmingly blamed Russia (figure 4). The largest change was in Eastern Ukraine. Individuals with Russian nationalities were more likely to blame the USA/NATO/Ukraine for the tension between Russia and Ukraine before the invasion. After the war began, both Natives and Russians shifted their blame towards Russia substantially (figure 2). Likewise, in Ukraine before the invasion, Russian speakers blamed the USA/NATO/Ukraine more than people who could only read or communicate in Russian (figure 2). People fluent in Russian shifted their blame to Russia after the war began. Figure 3 shows the same interactions as in figure 2, but in terms of opinion as to whether Russian policy benefits the country or hurts the country. Again, the shift is towards unanimity in Ukraine, compared with little change in Belarus before and after the invasion (figure 3).

In addition to our analysis of individual demographic groups, we collapsed all of the demographic variables captured in the survey into two-dimensional space using Principal Component (PC) Analysis (using *FactoMineR* and *Factoextra* in R v. 4.3.1). As identity often consists of multiple demographic attributes, summarizing the demographic data using PCA allows us to visualize changes across demographic profiles rather than individual attributes. The first PC explains 7.6% and 5.9% of the variance from Belarus and Ukraine, respectively. We label these similar PCs as *cosmopolitanism* because, in each country, the loadings on PC1 reflect young, secular, educated, middle-class people who use Western social media. More negative values of PC1 indicate older Orthodox, poor pensioners who watch national or Russian mass media. The second dimension from the Belarus PCA explains 4.4% of the variance and is best described as *social marginalization*. More positive values indicate young, poor, Polish, Muslim or Catholic workers. More negative values indicate people who are well established within Belarus, like those who are educated, Russian, Orthodox/secular, or have management/professional positions. The second dimension for Ukraine explains 4.6% of the variance and can be defined as *conscription vulnerability*, meaning people who are most likely to fight in the war. The more positive values indicate young, poor students who have not completed their higher education. More negative values indicate older, wealthy professionals.

**Figure 5.**
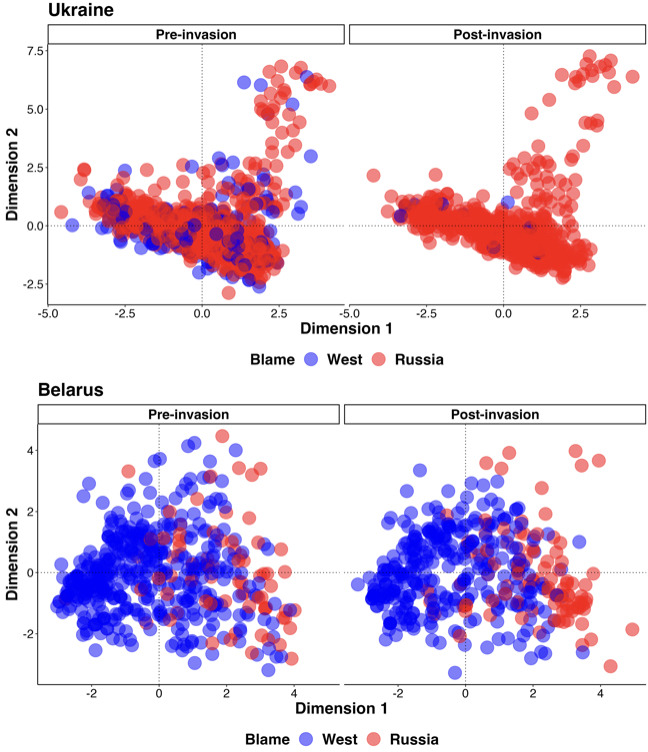
First two dimensions from the PCAs of (top) Ukraine and (bottom) Belarus. The first and second dimensions for Ukraine are cosmopolitanism and conscription vulnerability, respectively. For Belarus, they are cosmopolitanism and social marginalization.

Figure 5 shows the distribution of respondents across these dimensions coloured by those who blame Russia or the West for the tension/war in Ukraine pre- and post-invasion. These results reflect the same findings from our ANOVA analysis above. Namely, across demographic profiles in Ukraine, blame uniformly shifts towards Russia after the invasion (change from blue to red in figure 5*a*). While in Belarus, this shift is less pronounced. Again reflecting our results above, of the little shift in blame that happened in Belarus, respondents who were higher in dimension 1 (more secular, educated and middle-class) were more likely to blame Russia for tensions, contrasting the shift across all demographic groups in Ukraine.

Finally, we considered whether the shift in Ukraine could have been caused, in theory, by the change in our random survey sample in Ukraine due to the estimated 6 million Ukrainians (15% of the population) emigrating after the start of the war. To estimate how much difference this could make, we removed 15% of the most pro-Russian individuals from the first wave of our survey and compared this new ‘post-immigration’ sample to the original wave 1 sample. This did not change our results, confirming that emigration would not have explained the shift in attitudes we observed in Ukraine.

## 4. Discussion

**Figure 6.**
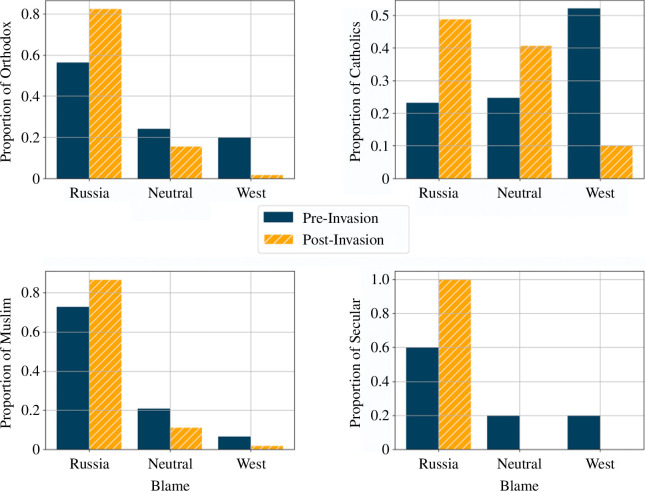
Distribution of blame attribution within each religious subgroup in Ukraine before and after the invasion.

Overall, in Ukraine but not in Belarus, the invasion (*season*) significantly interacted with the primary effects of region, nationality, and Russian language proficiency (all p<0.001 in tables 4 and 5). In Ukraine, there was some heterogeneity in how this shift occurred across religious groups. Looking more closely, we see that in Ukraine before the invasion, Orthodox people blamed the USA/NATO/Ukraine for tension more than any other religion (figure 2). In Ukraine, the effect within religion (figure 6) was not as strong as other demographics because, while there was a full shift among Orthodox Ukrainians, about half of Ukrainian Catholics post-invasion still blamed the West (10%) or were neutral (40%). Muslims blamed Russia more than Catholics as well. Both Catholics and Orthodox people shifted their blame to Russia after the war began (figure 2).

In contrast, in Belarus, the primary effects of the majority of demographic groups, including nationality, language and religion, were not significantly moderated by the invasion. The only primary effects significantly moderated by the invasion were the region in which a participant resided in and their education level (both p<0.05 in table 4), and these were only subtly moderated. Respondents with higher education were more likely to blame Russia than people with general or advanced secondary education levels. Also, people with higher education were more likely to blame Russia after the war began. Although Belarus is a dictatorship, which lessens press freedom to influence public opinion, there was still regional variation, as people in Minsk blamed Russia the most, before and after the invasion of Ukraine (table 4).

Our nationwide survey samples, from both Ukraine and Belarus in spring and summer 2022, document the change in identity-related attitudes in Ukraine after an invasion event. Our study sheds light on what Ukrainians believe about who is to blame for the war—before and after its beginning. After the invasion, the increase in blame towards Russia—in Ukraine but not in Belarus—is consistent with expectations for shared awareness [26,27] and shared dysphoria and threat [28,36–38].

The abruptness and magnitude of the shift in attitudes in Ukraine after the 2022 invasion are exceptional. In our studies done before the invasion, respondents in both Ukraine and Belarus had prioritized aspects of social group identity, with ethnic, religious and linguistic identities in Ukraine predicting moral attitudes of who is good and who is bad, who is to blame and who is the victim [35]. For decades, diverse social identities have persisted in Ukraine [39–41], a country that has historically and politically been torn between Russia and Europe [42–44]. Until Russia’s invasion of Ukraine, the Russian Orthodox Church, for instance, maintained a formidable presence through the Ukrainian Orthodox Church. After the 2014 Maidan revolution and Russia’s annexation of Crimea, there were still segments of the Ukrainian population, especially in the southeastern regions of Ukraine, whose affinity towards Russia remained strong [45,46]. Prior to the 2022 invasion, even certain Western scholars maintained that the West and its policies were to blame for the conflict [47].

## 5. Conclusion

Threat and violence lead to political solidarity [6,48] through shared purpose and mutual protection, with social identity distinctions being downplayed [49,50]. Identifying with a given ethnic, religious and/or linguistic group aligns an individual’s psychology and behaviour to the prevailing social norms of that group. The interplay between risk perception and social identity is context-dependent [51] and often affected by political and social allegiances [52].

Our results have revealed how an abruptly nationalized war, prompting in-group cohesion, transcended the previously considerable psychological power of ethnic, religious and linguistic identities. Although usually social identities are powerful drivers of human beliefs, emotions and behaviours, our observations provide nation-scale evidence that shared dysphoric experience of collective threat and the tightening of social norms [3,4,28] can override longstanding, deep-seated divisions among these identities.

## Data Availability

The processed data used to produce all our results are available online [53].
